# Editorial: Imaging in structural heart interventions

**DOI:** 10.3389/fcvm.2024.1364243

**Published:** 2024-02-14

**Authors:** Martijn G. H. Vrijkorte, Omar K. Khalique, Stamatios Lerakis, Martin J. Swaans

**Affiliations:** ^1^ Cardiovascular Imaging Department, St. Antonius Hospital, Nieuwegein, Netherlands; ^2^ Division of Cardiology, Department of Medicine, Structural Heart and Valve Center, Columbia University Medical Center, New York, NY, United States; ^3^ Mount Sinai Fuster Heart Hospital, New York, NY, United States

**Keywords:** structural heart interventions, TAVI, multimodality imaging, echo, fusion, ICE, tricuspid interventions, MRI

**Editorial on the Research Topic** Imaging in structural heart interventions

The field of transcatheter interventions for structural heart disease is rapidly advancing. In response to an aging population there is an increasing demand for percutaneous solutions. Especially in valvular heart disease, which is increasingly common among older individuals facing a prohibitive surgical risk, transcatheter approaches are becoming the preferred choice.

Over the past two decades, there have been significant advancements in transcatheter procedures, particularly in the aortic and mitral domains. The ongoing refinement in devices and techniques has significantly improved the outcomes in this patient population. This progress has now also expanded into the tricuspid domain.

Amidst this evolution in structural heart interventions, the pursuit of safety and efficacy has driven the exploration of cutting-edge imaging techniques, for example 3D echocardiography, 3D IntraCardiac Echocardiography (ICE) and fusion imaging. Nonetheless, the significance of multimodality imaging remains crucial in the context of structural heart disease.

This series of articles delves into the crucial aspects of multimodality imaging during transcatheter structural heart interventions, such as pre-procedural planning and procedural guidance.

The article by Ho et al. focusses on the adjunctive role of 3-dimensional (3D) ICE in structural heart interventions. The role of 3D ICE in various percutaneous interventions was explored, including transcatheter mitral and tricuspid interventions, atrial septal defect closure, and paravalvular leakage closure. The workflow considerations, challenges, and limitations associated with the adoption of 3D ICE are acknowledged. The comparison between 3D ICE and 3D TransEsophageal Echo (TEE) is also explored, concerning the differences in matrix array size, spatial and temporal resolution, and optimal imaging planes. 3D ICE is especially useful in situations where technical challenges arise with TEE.

The case report by Medda et al. presents a “snaring-assisted” TAVR procedure for severe Aortic Stenosis (AS). Pre-operative CT showed severe calcific AS with a porcelain aorta and a gothic aortic arch. The authors successfully performed the TAVR procedure under complete cerebral protection (CP). Advancement and delivery of the valve was performed “snaring-assisted” with the “Chaperone” and “Top Hat” technique to avoid interference with the CP device and calcified aortic arch. The technique facilitated an atraumatic advancement of the catheter resulting in a successful implantation without complications. Macroscopic assessment of the CP device showed small debris of calcium.

The pilot study by Bertsche et al. evaluated non-contrast-enhanced 3D Cardiac Magnetic Resonance imaging (CMR) for preprocedural planning of percutaneous Left Atrial Appendage closure (LAAc). Currently, TEE and fluoroscopy (XR) are the golden standard, but with the limitation of exposure to ionizing radiation and contrast. The authors investigated CMR as an alternative for quantification of LAA dimensions and determining optimal C-arm angulation for LAAc.

The study involved 13 patients prior to LAAc. CMR-derived measurements of the landing zone (perimeter and area) showed excellent congruency with periprocedural XR. However, the maximum diameter from CMR tends to overestimate compared to XR, likely caused by limited spatial resolution in CMR and non-optimal XR angulation. CMR-based measurements also showed a high correlation with TEE-based measurements. Furthermore, CMR predicted angulation and periprocedural used angulation were in good accordance in 85% of cases.

The meta-analysis by Lan et al. compared the efficacy and safety of ICE vs. TEE for guiding transcatheter closure of both Atrial Septal Defects (ASD) and Patent Foramen Ovale (PFO). The authors incorporated data from 11 studies, involving 4,748 patients. ICE was associated with significantly shorter Fluoroscopy Time (FT) and Procedure Time (PT) compared to TEE, with similar success rates for complete closure. Patients guided by ICE also had a shorter hospital stay and ICE demonstrated a lower incidence of adverse events, including arrhythmias and vascular complications. This study showed that ICE can reduce radiation exposure, and can eliminate the need for general anesthesia in ASD and PFO closure, without an increase in global costs.

Koren et al. introduces a predictive model for Iliofemoral Vascular Complications (IVC) in TransFemoral Transcatheter Aortic Valve Replacement (TF-TAVR). IVC remains a significant issue, affecting nearly a quarter of patients. The study aims to create a risk score for calcified and tortuous vessels, using anatomical parameters from a retrospective analysis of 3,119 TF-TAVR patients. CT images from 516 matched patients were used to establish a predictive model, validated with a cohort of 609 patients. Predictors for IVC include Sheath-to-Femoral Artery diameter Ratio (SFAR), Sum Of Angles (SOA), Number Of Curves (NOC), and Minimal Lumen Diameter (MLD). The indexed risk score (CSI), incorporating SOA, NOC, and MLD, exhibits strong sensitivity and specificity. In the final model, including SFAR >1.00 and the CSI set to >100, sensitivity and accuracy was further improved and resulted in a robust model for IVC risk stratification in TF-TAVR.

The review by Abdelshafy et al. explored the use of quantitative aortography by Video-Densitometry (VD) to evaluate the Regurgitant Fraction (RF) after Transcatheter Aortic Valve Replacement (TAVR), focusing on ParaValvular Leak (PVL). Although TransThoracic Echo (TTE) is safe and the preferred option for assessing PVL, the lack of consistency poses a limitation. Angiography enables immediate post-procedural PVL grading with the sum of all jets, and significant PVL can be corrected with post-dilatation.

The authors suggest an Left Ventricular Outflow Tract-Aortic Regurgitation (LVOT-AR) of 17% as the optimal threshold to distinguish in AR severity. This correlated strongly with intra-procedural TEE. This was further confirmed with CMR-derived RF for PVL quantification. LVOT-AR can also serve as a tool to guide decision making for Balloon Post-Dilatation and can offer insights into the performance of different devices.

The review by Tomlinson et al. provides a comprehensive overview of the landscape of Transcatheter Tricuspid Valve Interventions (TTVI), emphasizing the pivotal role of multimodality imaging. This article describes protocols and methodologies employed in the multimodality assessment of TR. It aims to provide insight into the pathophysiology of TR, and anatomical and functional characteristics, critical for appropriate device selection.

Moreover, intra-procedural imaging recommendations are presented, including Transcathter Edge-to-Edge Repair (TEER), annuloplasty, orthotopic valves, and heterotopic valves. All relevant imaging modalities are being addressed for each particular device, emphasizing the role of 3D rendering and MultiPlanar Reconstruction (MPR).

**Figure 1:**
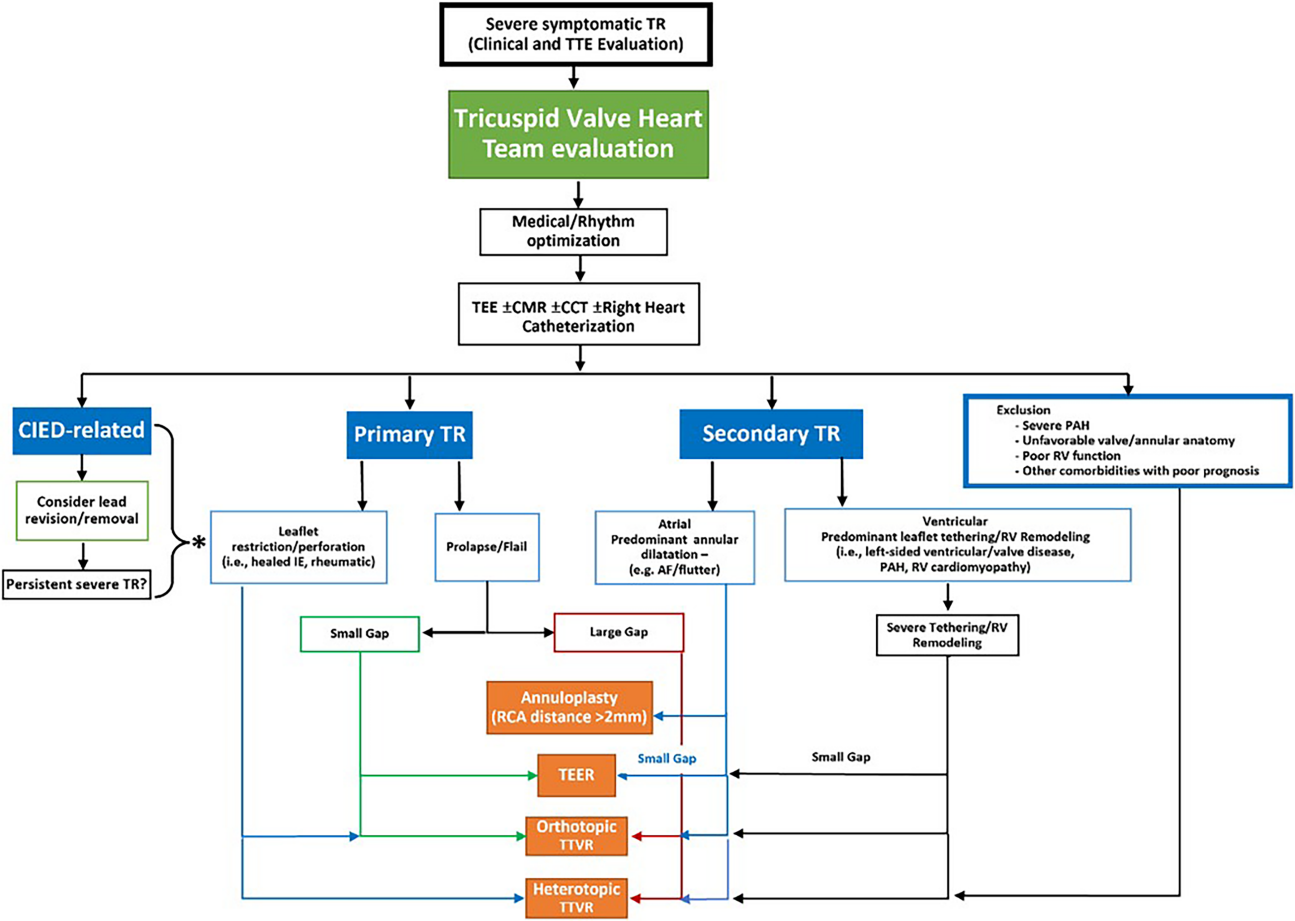
Proposed workflow algorithm for device class selection for transcatheter tricuspid valve intervention. TEE, transesophageal echocardiogram; CMR, cardiac magnetic resonance imaging; CCT, cardiac computed tomography; CIED, cardiac implantable electronic devices; TR, tricuspid regurgitation; PAH, pulmonary arterial hypertension; RV, right ventricular; IE, infective endocarditis; AF, atrial fibrillation; RCA, right coronary artery; TEER, transcatheter edge to edge repair; TTVR, transcatheter tricuspid valve replacement.

The authors also provided a workflow algorithm for adequate device selection (Figure 1).

